# Endovascular Treatment of Stroke Caused by Carotid Artery Dissection

**DOI:** 10.3390/brainsci10110800

**Published:** 2020-10-30

**Authors:** Grzegorz Meder, Milena Świtońska, Piotr Płeszka, Violetta Palacz-Duda, Dorota Dzianott-Pabijan, Paweł Sokal

**Affiliations:** 1 Department of Interventional Radiology, Jan Biziel University Hospital No. 2, Ujejskiego 75 Street, 85-168 Bydgoszcz, Poland; 2 Stroke Intervention Centre, Department of Neurosurgery and Neurology, Jan Biziel University Hospital No. 2, Ujejskiego 75 Street, 85-168 Bydgoszcz, Poland; m.switonska@cm.umk.pl (M.Ś.); pio.ple@wp.pl (P.P.); violkapduda1@tlen.pl (V.P.-D.); 3 Department of Neurosurgery and Neurology, Faculty of Health Sciences, Nicolaus Copernicus University in Toruń, Ludwik Rydygier Collegium Medicum, Ujejskiego 75 Street, 85-168 Bydgoszcz, Poland; pawel.sokal@cm.umk.pl; 4 Neurological Rehabilitation Ward Kuyavian-Pomeranian Pulmonology Centre, Meysnera 9 Street, 85-472 Bydgoszcz, Poland; dorota.dzianott-pabijan@wp.pl

**Keywords:** stroke, artery dissection, endovascular treatment, stenting, mechanical thrombectomy

## Abstract

Ischemic stroke due to large vessel occlusion (LVO) is a devastating condition. Most LVOs are embolic in nature. Arterial dissection is responsible for only a small proportion of LVOs, is specific in nature and poses some challenges in treatment. We describe 3 cases where patients with stroke caused by carotid artery dissection were treated with mechanical thrombectomy and extensive stenting with good outcome. We believe that mechanical thrombectomy and stenting is a treatment of choice in these cases.

## 1. Introduction

Ischemic stroke due to large vessel occlusion (LVO) is a devastating condition, bearing great risk of severe disability and death [1]. Most LVOs are embolic in nature. Although only a small proportion of all LVOs arise from an arterial dissection, it accounts for approximately 20% of strokes among individuals under the age of 45 [2,3]. While some dissections can be caused by traumatic arterial injury, in most cases, dissection is considered to be idiopathic. There are some conditions that can predispose to dissection such as atherosclerosis, hypertension, Marfan syndrome, and fibromuscular dysplasia [4]. Managing stroke caused by carotid dissection can be challenging. In this report, we present a series of three cases of patients with stroke due to acute extracranial carotid artery dissection that were admitted to our stroke centre and treated with mechanical thrombectomy and extensive stenting with good outcome. We believe that mechanical thrombectomy and stenting can be a treatment of choice in these cases.

## 2. Case Presentation

**Figure 1:**
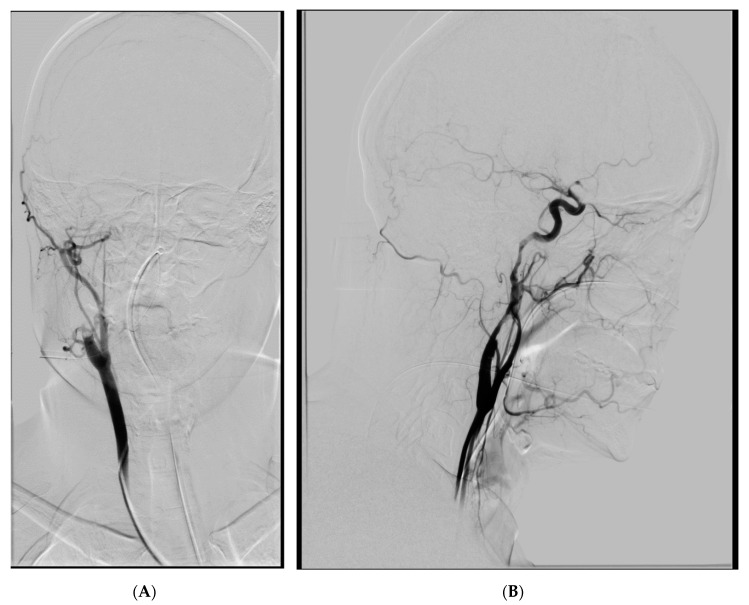
(**A**) Thrombosis and dissection of the right ICA, AP view. (**B**) Extent of the dissection, lateral view.

The first patient was a 34-year old woman, with no previous drug and medical history, involved, as a passenger, in a car accident a few hours before stroke symptoms appeared. After the accident, she was an Emergency Room (ER) patient in a local hospital undergoing observation and a series of tests: trauma computed tomography (CT) scan, X-ray scans etc., which only revealed fracture of the sternum, face, and scalp bruises. After a few hours, being in a well and stable condition, she suddenly deteriorated and presented with dense left-sided weakness. She was immediately transferred to the CT suite and non-contrast CT scan of the head was performed followed by CT angiography of the neck and head. The CT scan of the head, apart the aforementioned face and scalp bruises, was normal, while the CT angiography revealed right middle cerebral artery (MCA) and Internal Cerebral Artery (ICA) occlusion with probable carotid dissection as an underlying cause. The patient was transferred to our stroke centre for endovascular treatment. During diagnostic arteriography, we confirmed dissection and thrombosis of the right carotid artery in segments C1–C2 (Figure 1A).

**Figure 2:**
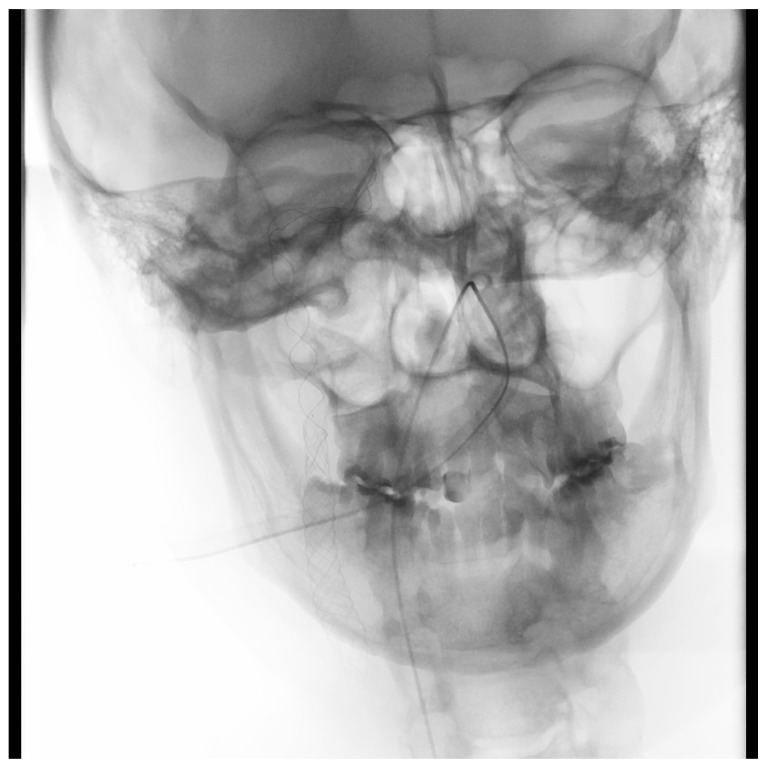
Stented right ICA (non DSA image).

The first treatment goal was to remove the thrombus and open the artery. We used, a 8F short introducer sheath in the groin and then placed a long neuro sheath (6F NeuronMax, Penumbra Inc., Alameda, CA, USA) in the common carotid artery. Through the long sheath, an aspirating system (ACE68, Penumbra Inc.) was introduced into the ICA and the large thrombus was removed by aspiration only. The extent of the dissection and ICA-T occlusion were visualized (Figure 1B). With the microcatheter (Rebar 18, Medtronic, Dublin, Ireland) and J-shaped microguidewire (Hybrid 1214, Balt Extrusion, Montmorency, France), the true lumen of the dissected artery was found and catheterized to the point above the visible dissection (C3). In the next step, the ACE68 catheter was gently pushed over the Rebar microcatheter to the ICA terminus and then over the microguidewire, we pushed the Rebar microcatheter into the M2 segment of the MCA. In the next step, we used a stent-retriever (Catch V20, Balt Extrusion) and with continuous aspiration from the ACE68, we removed the clot from MCA-ICA, leaving the aspiration catheter in place. The last steps of the procedure consisted of placing three intracranial self-expanding braided stents (Leo+, Balt Extrusion) in a telescopic manner starting from the C3 ICA segment and moving caudally to the C1 segment and at the end, a braided carotid stent (Roadsaver, Terumo, Tokyo, Japan) was placed to reach the CCA (Figure 2). After placing the stents, we performed an internal massage maneuver with the microcatheter and then balloon angioplasty of the stents using a 5 mm compliant intracranial balloon (Copernic, Balt Extrusion) in the ICA C1-C3 segments, a 5 mm PTA balloon (Submarine, Medtronic) in ICA C1 and a Roadsaver stent to obtain optimal stents apposition. During the procedure, the patient was given 5000 units of heparin through intravenous (IV) administration and a weight-based bolus of eptifibatide. After the procedure, the patient remained in the angio-suite for 30 min, during which time we performed three check-runs for early stent thrombosis. The patient was then transferred to the neurologic intensive care unit for 1 day with continuous eptifibatide IV; 4 h before it was completed, she was started on oral dual antiplatelet therapy: ticagrelor 90 mg twice a day and acetylsalicylic acid (ASA) 75 mg once a day. She was in our hospital for 25 days, undergoing early neuro-rehabilitation with good clinical improvement: NIHSS 15→8 (National Institute of Health Stroke Scale). During the stay, we observed transient 1-day haematuria, which resolved without treatment. After 25 days, the patient was in stable condition modified Rankin Scale (mRS): 4/3 and transferred to the rehabilitation unit. After 3 months, she was independent, walking and had minor left-hand weakness (mRS 1). Control CT angiography showed patent stents in the right ICA and patent intracranial arteries.

**Figure 3:**
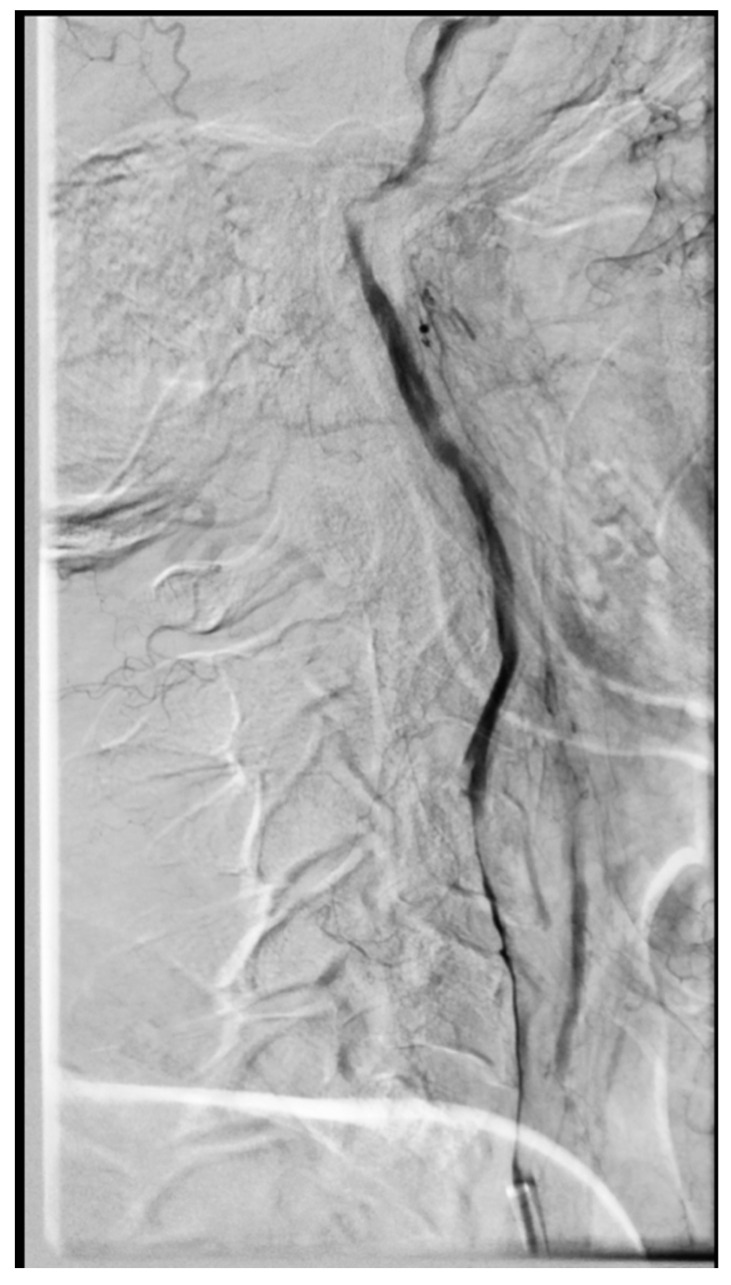
Right ICA, long dissection.

The second patient was a 62-year old male, with a medical history of smoking and arterial hypertension—treated with lercanidypine and telmisartan. He was driving a truck when neurological symptoms associated with the right brain hemisphere (dense left-sided weakness) occurred, leading to a minor car accident. The patient was transferred to our hospital by a paramedic team. A CT scan performed at ER revealed occlusion of the right ICA and hyperdense right MCA. During diagnostic angiography, we confirmed right ICA occlusion, starting from the C1 segment. Using the same technique as described for the previous patient, we aspirated a large clot from the ICA, which allowed us to find a long ICA dissection (Figure 3).

**Figure 4:**
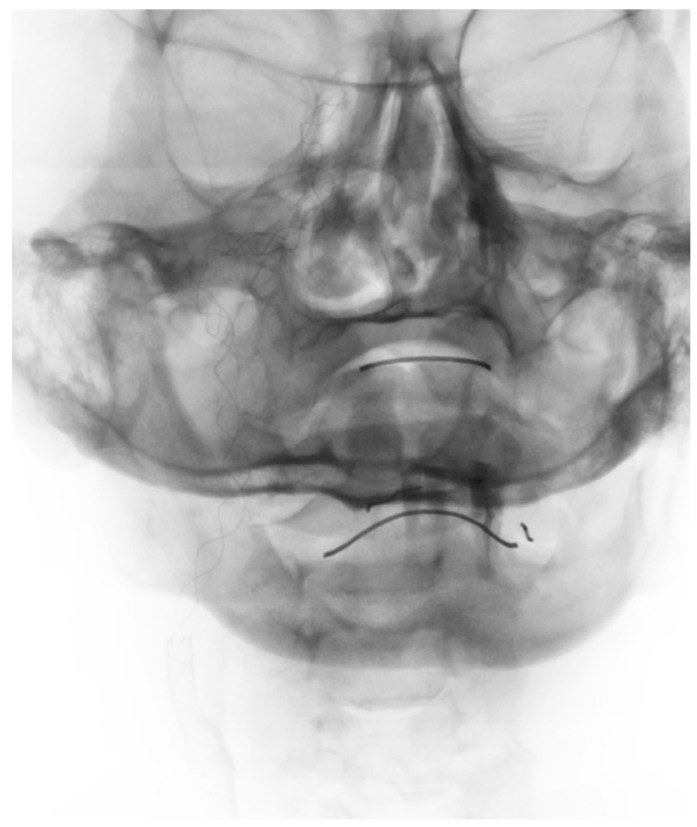
Four overlapping stents covering dissected ICA segments (non-subtracted image).

After removing the clot from the ICA, we performed mechanical thrombectomy of the MCA and implanted four overlapping stents covering the dissected ICA segments (Figure 4). In this case, we also used braided intracranial stents (Leo+) and the last stent covering the CCA-ICA junction was a typical carotid laser-cut stent (Protégé, Medtronic). During the procedure, the patient was given IV of a weight-based bolus of eptifibatide and 5000 units of heparin, but we observed early clot formation at the top of the first implanted stent (C3) during the first control run. The clot was removed with an aspiration catheter (ACE68, Penumbra). After a 30-min observation with control angiographies every 10 min, the patient, without sequelae of the clot formation, was transferred to the neurology department where he underwent early rehabilitation, improving quickly (NIHSS 17→5). After 7 days, he was released to go home with no significant disability (mRS 1). Control CT angiography showed patent stents in the right ICA and patent intracranial arteries.

**Figure 5:**
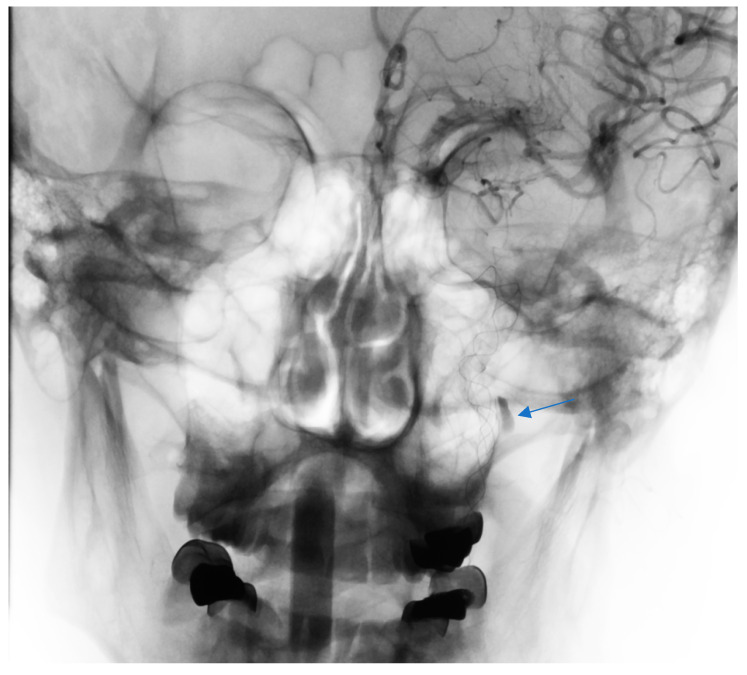
Stented left ICA with 2 overlapping stents covering the dissection and pseudoaneurysm with contrast medium stagnation (arrow) in late phase.

The third patient was a 56-year old male, with no previous medical and drug history, who suffered from a spontaneous left ICA C1 dissection with total ICA occlusion, pseudoaneurysm, and left MCA occlusion resulting in large left hemisphere syndrome, NIHSS 18 at admission. Using the technique described above, we removed the clot from the ICA and then from the MCA and stented the dissected ICA segment using 2 braided intracranial stents (Leo+, BALT) with good result (Figure 5). After 3 months of rehabilitation, the patient was discharged in stable condition, walking with minor aphasia and mild right arm weakness (mRS 2). We were unable to reach this patient for follow-up control CT angiography as he was a citizen of a foreign country and returned home after treatment.

## 3. Discussion

Arterial dissection is characterized by the penetration of blood into the arterial wall through a tear, which develops in one or more layers of the wall. If the tear is shallow, only the intima may be dissected, which can lead to the presence of an intimal flap with or without formation of the thrombus inside the true and false vessel lumen. Deeper tears of the arterial wall may lead to a sub-adventitial dissecting aneurysm or even transection of the arterial wall and lethal bleeding [4,5].

Most dissections occur spontaneously. The second most common causative factor is head or neck trauma, especially in car accident victims. Reported trauma can sometimes be very minor or even anecdotal like sudden head turns, playing golf, jumping or chiropractic maneuvers [4,5].

Severity of the disease, anatomical features, and potential complications are well characterized by the classification proposed by Colorado University [6,7] (Table 1). Apart from lethal bleeding due to artery transection, the second most serious and common complication of artery dissection are ischemic in nature. Although artery dissection is a rather uncommon cause of stroke in the general population, it accounts for 20% of strokes in patients under the age of 45. Those strokes are caused by thrombus formation and either occlusion or narrowing of the vessel lumen. Clots originating from the dissected artery segments may also migrate distally to cerebral arteries causing secondary occlusions.

The diagnosis of dissection is based on radiologic findings in ultrasound, CT, computed tomography angiography (CTA), magnetic resonance imaging (MRI), and magnetic resonance angiography (MRA); however, digital subtraction arteriography (DSA) is still considered to be the gold standard [5]. Typical radiologic findings include: narrowed and irregular vessel lumen, tapered ICA occlusion with the carotid bulb spared, double lumen appearance, pseudonaneurysm, intimal flap, or eccentric thickening of the arterial wall. In our experience, recognizing dissection as the cause of stroke is quite easy in young patients with trauma but may be challenging in older patients with unknown medical history and total artery occlusion as the radiologic features of the artery dissection may sometimes mimic tandem occlusions or massive emboli.

There is no clear consensus regarding management of dissections. Grade V is considered to be lethal. It is widely accepted that low grade [1] dissections with no bleeding and minor or transient ischemic symptoms can be treated with 3–6 months of antithrombotic therapy with anticoagulant or antiplatelet agents [8,9,10]. To our understanding, as the pharmacologic treatment of higher-grade dissections carries a very low success rate, the approach in these cases should be more aggressive i.e., surgical or endovascular [11,12]. In the case of stroke symptoms with intraluminal thrombus formation such as total artery occlusion or distal emboli, the only viable option seems to be prompt endovascular treatment.

The nature of the condition must indicate the type of endovascular treatment. The first goal is the same as in typical embolic stroke—to remove the clot within the time window advised for mechanical thrombectomy [13]. After this, further (especially intracranial) progression of the dissection should be prevented, and the artery should be kept patent.

There are two main methods of retrieving a thrombus from an artery: aspiration or using stent retrievers [14]. In our opinion, it is advisable to not introduce metal devices such as stent retrievers into the dissected artery segment in order to prevent vessel walls from incurring further damage. Moreover, if the artery is completely occluded, there is no clear answer as to whether the thrombus is inside the true or false lumen. In these cases, we always use aspiration first, which seems to be less traumatic and suction power can be adjusted. While it may be debatable in the cases described here whether the thrombus was aspirated from the true or false lumen, it is important to remove it in its entirety, or as much of it as possible. Suction applied to the false vessel lumen may even be beneficial, leading to its collapse and opening of the true lumen. Revealing the true vessel lumen is of great importance as it allows for the visualization of the extent of the dissection and potential distal emboli.

In two of the described cases, patients had thrombi in the intracranial arteries. Removing such clots requires placing catheters in the true vessel lumen. Catheterization may sometimes be difficult and has to be done gently to avoid progression of the dissection. We strongly suggest using a microcatheter with a J-shaped microguidewire placed in a large lumen aspiration catheter and confirm its advance and placement by injecting contrast into the microcatheter. Passing the intracranial thrombus with the microguidewire and microcatheter allows you to use a combination of a stent retriever and aspiration, which seems to be the most efficient method [15,16]. During clot removal, we leave the aspiration catheter in place to reduce the number of passes through the dissected segment of the artery. After removing the clot from intracranial arteries, you can progress to the next step, which involves placing stents to keep the artery patent.

Final confirmation of proper microcatheter placement is mandatory before stent placement. The technique used in the cases described here consists of placing intracranial braided stents in a telescopic manner starting from the top of the dissected segment and moving caudally to cover the whole damaged portion of the artery. If there is a need for microcatheter exchange, it should be done carefully with the long microguidewire left in place first in order to prevent losing the access route. We found that the intracranial braided stents made by Balt suited our needs best due to the largest diameter available being up to 5.5 mm, and longest length being up to 75 mm; this helps to reduce the number of necessary maneuvers and stents implanted. The radial force of the stent seems to be sufficient to push the damaged intima back against the vessel wall, especially when the false vessel lumen is collapsed after aspiration. In the case of dissections starting from the internal carotid origin, the last stent we used was a carotid stent, either braided or laser cut. In the final step, we performed balloon angioplasty to achieve the best possible stent apposition. Angioplasty is performed also from the top to the bottom using intracranial remodeling balloons at first, and then high-pressure balloons in the carotid or intracranial stents placed in the C1 segment if needed.

Placing stents in the acute phase requires a prompt introduction of antiplatelet therapy to avoid early in-stent thrombosis. In the cases described here, we administered 5000 IU of heparin before the first stent was placed, and patient weight-calculated (180 mcg/kg) bolus of GP IIb/IIIa inhibitor-eptifibatide. We prefer unfractionated heparin to the low molecular weight heparin (LMWH) such as enoxaparin, since in the case of intraprocedural bleeding, the effect of unfractionated heparin can be reversed by administration of protamine sulphate. After the procedure, GP IIb/IIIa inhibitors were infused continuously (2 mcg/kg/min) for 24 h. In emergency cases of stenting such as those described in this paper, we use GP IIb/IIIa inhibitors as the first line of antiplatelet treatment—as intravenous administration of these drugs, provide almost instant platelet inhibition. Of the GP IIb/IIIa inhibitors we mostly choose eptifibatide since its effect is reversible and platelet function returns towards baseline within 4 h after stopping a continuous infusion.

Oral antiplatelets (300 mg of ASA and 180 mg of ticagrelor) were introduced 4 h before the end of the GP IIb/IIIa inhibitors’ infusions. Oral dual antiplatelet therapy was continued for the next 3 months: 75 mg of ASA once a day with 90 mg ticagrelor twice a day, and after that 75 mg of ASA once a day for one year was prescribed. We have chosen a combination of ASA and ticagrelor because of their efficacy and safety profile [17]. Introducing anticoagulants and antiplatelets in patients with stroke and emergency stenting has been widely discussed, and although there are currently no clear guidelines regarding their use, recent papers show the safety of this approach [18,19,20,21,22].

Large extent of the brain ischemia prior to endovascular treatment (EVT) is a known predictor for both intracranial hemorrhage (ICH) and later poor clinical outcome in patients with stroke [23,24,25]. In our institution, we use widely accepted 10-point Alberta Stroke Program Early CT score (ASPECTS) to assess the ischemic changes on a non-contrast head CT before treatment and avoid mechanical thrombectomy (MT), in patients with ASPECTS < 6 and MT combined with stenting in patients with ASPECTS < 7 [25]. The first two described patients were treated early (less than 3 h) in the time window for mechanical thrombectomy and had high (9–10) ASPECTS before the treatment. In control CT, which is made in every patient 24 h after EVT, they developed only moderate ischemic changes in the affected MCA territories, measuring 4 cm in diameter for the first patient and 3 cm for the second one. The third patient was treated in the 5th hour from the symptoms’ onset, with initial ASPECTS 7. In control CT, he developed large left MCA territory edema with slight 5 mm midline shift, both of which decreased significantly after 7 days of symptomatic treatment with mannitol and diuretics. In all cases, we achieved complete reperfusion during EVT and none of the patients developed ICH after EVT despite the introduction of antiplatelet drugs. Still, it is worth noting that caution should be taken when administering these medications to patients treated towards the end of the time window for MT, with low initial ASPECTS, incomplete reperfusion and especially in the case of trauma patients as it may trigger potential bleeding [23,26,27]. For each individual case, the expected benefits should outweigh the risk. We only noticed one event of bleeding, which occurred 3 days post-treatment as an episode of mild haematuria in the first patient and it resolved with no intervention and without sequelae. This patient had a full body trauma CT scan performed after the accident, which had been evaluated by two experienced radiologists and any active internal bleeding had been excluded. She also had another CT scan of the abdomen and pelvis after the episode of bleeding, showing the same result.

## 4. Conclusions and Future Directions

Mechanical thrombectomy and extensive artery stenting can be beneficial in patients with acute, high grade extracranial carotid artery dissections with artery occlusions and distal embolic complications. Medical consensus is lacking regarding ideal therapeutic strategy in those cases. Results of randomized-controlled trials like ongoing TITAN (Thrombectomy In TANdem lesion) (NCT03978988) will probably help to determine potential benefits and risk associated with acute stent placement and periprocedural antiplatelet therapy. Additionally, recent advances in vascular biology such as using microvessel-on-chip can potentially help to study in vitro the best practice regarding the use anti-coagulants and determine their optimal dose using humanized models [28,29].

## Figures and Tables

**Table 1:** Denver dissection grading system in blunt cerebrovascular injury and associated stroke rates.

Grade	Type of Dissection	Stroke in Carotid Artery Dissection (%)	Stroke in Vertebral Artery Dissection (%)
I	Luminal irregularity or dissection with < 25% luminal narrowing	3	19
II	Intimal flap or intramural hematoma with luminal narrowing ≥ 25% or intraluminal thrombus	11	40
III	Pseudoaneurysm	33	13
IV	Total occlusion	44	33
V	Transection and free bleeding	100	N/A

## Abbreviations

*CT*	Computed tomography
*DSA*	Digital subtraction arteriography
*ER*	Emergency Room
*GCS*	Glasgow Coma Scale
*mRS*	modified Rankin Scale
*ICA*	Internal carotid artery
*ICU*	Intensive Care Unit
*MCA*	Middle cerebral artery
*MRA*	Magnetic resonance angiography
*MRI*	Magnetic resonance imaging
*NIHSS*	National Institute of Health Stroke Scale
*ICA segments*	Bouthillier classification (C1–C7)
*LVO:*	Large vessel occlusion
*ASA*	acetylsalicylic acid
*MT*	mechanical thrombectomy
*EVT*	endovascular treatment
*LMWH*	low molecular weight heparin
*ICH*	intracranial hemorrhage
*ASPECTS*	Alberta Stroke Program Early CT score
